# Dr. Thanjavur Santhanakrishna Kanaka: Asia's First Female Neurosurgeon and a Pioneer of Functional Neurosurgery

**DOI:** 10.7759/cureus.69403

**Published:** 2024-09-14

**Authors:** Anshrutha Dhanashekar, Latha Ganti

**Affiliations:** 1 Research, Orlando College of Osteopathic Medicine, Winter Garden, USA; 2 Emergency Medicine and Neurology, University of Central Florida, Orlando, USA; 3 Medical Science, The Warren Alpert Medical School of Brown University, Providence, USA

**Keywords:** female neurosurgeon, functional neurosurgery, historical vignette, medical pioneer, neurosurgery

## Abstract

Dr. Thanjavur Santhanakrishna Kanaka significantly advanced Indian healthcare through her pioneering contributions to modern surgical techniques and advocacy for gender equity in medical education. Her research in stereotactic surgery and electrode implantation, coupled with her focus on cost-effective care for low-income patients, has had a profound impact on neurosurgery. Dr. Kanaka's enduring legacy continues to inspire and shape the field, reflecting her substantial influence on both medical practice and community service.

## Introduction and background

Historically, women have faced significant underrepresentation in medicine, especially in challenging surgical specialties such as neurosurgery [1]. In South Asia, this disparity was even more pronounced due to entrenched social barriers [2]. However, amidst these obstacles emerged trailblazers who shattered conventions and paved new paths. One such groundbreaking figure is Dr. Thanjavur Santhanakrishna Kanaka, Asia's first female neurosurgeon [3,4]. Completing her medical education and surgical residencies in Chennai, Tamil Nadu, Dr. Kanaka broke barriers with unmatched determination and passion. Her pioneering work in surgical techniques and deep brain stimulation (DBS) not only revolutionized neurosurgery in India but also set new standards across South Asia. This paper celebrates Dr. Kanaka's extraordinary achievements, her relentless fight for gender equity in medicine, and her transformative impact on healthcare and medical education.

## Review

Background and education

Dr. Kanaka was born on March 31, 1932, in Madras (now Chennai), Tamil Nadu, India, to a highly educated family, including seven siblings. Her father, Santhanakrishna, was the principal of Madras Teachers College and the Deputy Director of Public Instruction, while her mother, Padmavathi, stayed at home to raise the family [3,4]. Dr. Kanaka completed her early education in Chennai and was interested in pursuing spiritual studies from a young age. However, Dr. Kanaka's mother persuaded her to pursue medicine. Dr. Kanaka's parents were extremely supportive of her medical education, even during a time when India was undergoing so many changes as a country [4,5].

Dr. Kanaka completed her medical education at Madras Medical College (MMC) in the late 1940s, right around when India was becoming independent from British colonial rule. The "spirit of defiance" [3] from India's freedom fighters would follow Dr. Kanaka's approach to being a true pioneer of medical techniques and promoting female equality in the field throughout her lifetime and career. During her undergraduate years at MMC, in addition to her intense academic workload, Dr. Kanaka satiated her thirst for knowledge by being involved in several research projects, including using gonococcus bacteria to study leprosy, which was prevalent in India at the time, and investigating the properties of cerebrospinal fluid (CSF) [4].

Doctoral training and gender inequities

After graduating with an MBBS (Bachelor of Medicine, Bachelor of Surgery) in 1954, Dr. Kanaka wanted to go into a general surgery residency. However, the path to get to this point was quite tumultuous. Gender discrimination was the biggest hurdle for Dr. Kanaka, as surgery was male-dominated, both in India and throughout the world. Also, women were not encouraged to pursue higher education, especially in surgery. Indian families did not (and some still do not) encourage women to spend time in academics and getting a job but rather focused on the more "traditional" roles, such as cooking, cleaning, and taking care of a family, aspects of life that being a surgeon could strain pretty severely due to the long working hours and physical exertion [4]. Dr. Kanaka herself was told many times by the admissions committee that she would never get accepted. While these barriers were deterring, Dr. Kanaka's grit and determination allowed her to apply to the program. The entry point to the program relied on a written essay, and although Dr. Kanaka was academically gifted, the admissions committee would find faults and reject her application. Thus, it took multiple application cycles for Kanaka to be taken seriously. Only two other females were admitted to MMC's program in the past, one never practiced and the other became an anatomist [3,4].

After overcoming all of these obstacles, Dr. Kanaka was finally accepted to the Master of Surgery Program as the only female of a total of eight residents. While this achievement was massive, the hurdles Dr. Kanaka faced on the way would be a mark of her time as a resident. Gender discrimination was a huge issue in India at the time, and Dr. Kanaka definitely experienced her fair share as a resident.

Oftentimes, Dr. Kanaka was not allowed to scrub into surgical procedures because her supervisors were afraid to give her a scalpel to use, which led to her failing examinations during her program. They believed that she was not worthy of becoming a surgeon and the director of the program would conspire against her, making sure that she would not see any emergency cases or be a part of hospital visits. Dr. Kanaka would have to re-attempt the qualifying examinations multiple times, which was more than her male classmates, to show that she is capable of being a good doctor and surgeon. While India was still a new country at the time, the gender discrimination issues Dr. Kanaka faced during her training were not far unlike ones that are still prevalent in modern Indian medical training, even if to a lesser degree of harassment and unfair treatment.

**Figure 1:**
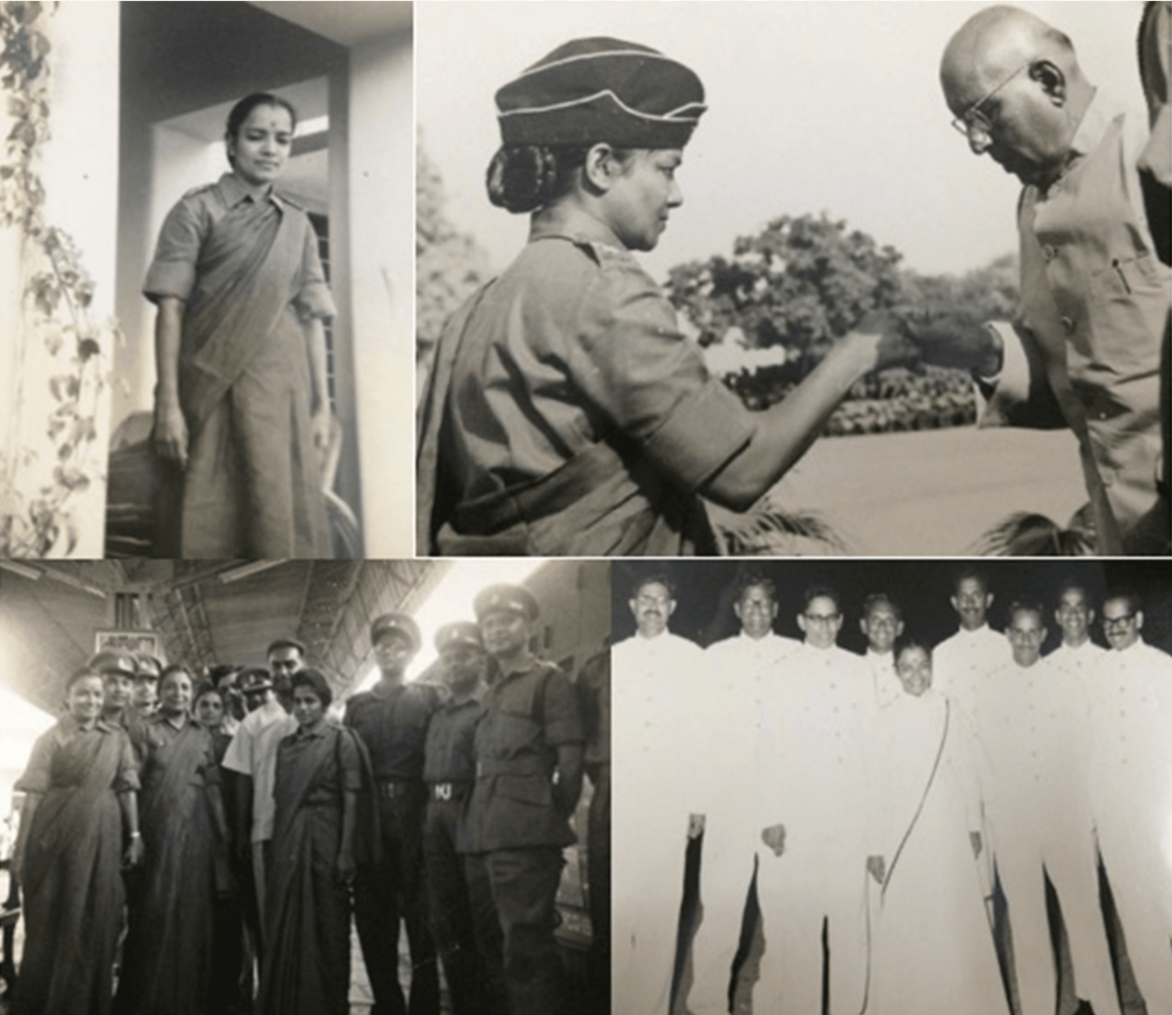
Photographs of Dr. Thanjavur Santhanakrishna Kanaka during her time serving in the Indian Army Reproduced with permission from Chidambaram S, Vasudevan MC, Pande A, Chidambaram S, Pannullo SC: Dr. Thanjavur Santhanakrishna Kanaka-a pioneer and neurosurgical innovator. World Neurosurg. 2021, 150:84-8. 10.1016/j.wneu.2021.03.079 [5]

A pioneer for change, Dr. Kanaka volunteered to help during the Indo-China war in 1962-1963 and was appointed as a captain in the Indian Army. She worked as a medical officer at the General Government Hospital in Chennai (Figure 1).

Dr. Kanaka's grit, perseverance, and determination truly helped her through a harrowing residency program, ultimately leading to her graduation in 1963 with a Masters in Surgery (MS) and a Masters in Neurosurgery (MS) in 1968. Her training was not entirely rough, as she had the support of a few mentors: Dr. Ramamurthi, a urologist she met during her undergraduate medical education, and Dr. A Venugopal [5].

Pioneering functional neurosurgery in India

The late 20th century saw a rise in new neurosurgery techniques around the world, one of which was stereotactic surgery (often just known as stereotax). Stereotactic surgery uses 3D imaging and many targeted laser beams not only to treat tumors but also to effectively deliver radiation to affected areas of the brain with minimal effects on surrounding tissue. The complex and precise method of stereotactic surgery does not involve true invasion methods, making it revolutionary in neurosurgery around the world [6]. During her time in neurosurgery residency, Dr. Kanaka spent a lot of time devoted to the hospital and became interested in such techniques. Alongside her mentors and other pioneers of neurosurgery in India, Dr. Kanaka became at the forefront of stereotactic surgery and functional neurosurgical techniques [5]. She pursued a PhD in stereotactic surgery, focusing on understanding its role in movement disorders, cerebral palsy, psychiatric disorders, and epilepsy, to name a few. After graduating in 1972 and obtaining a fellowship degree in 1980 from the University of California, Los Angeles (UCLA), Dr. Kanaka conducted over 1,700 stereotactic procedures in 15 years' time in her hometown of Chennai, India [7]. She produced a lot of papers regarding some of her notable findings, including her work on using surgical techniques for cerebral palsy [4,5].

In addition to stereotactic surgeries, Dr. Kanaka became a leader in using deep brain stimulation (DBS) techniques and chronic electrode implantation to help patients with epilepsy, cerebral palsy, and psychiatric disorders [5]. DBS involves the implantation of electrodes in the brain, at specific areas, and a battery in the body. The goal is to help improve the brain's electrical activity for patients with neurological disorders such as Parkinson's disease [8]. Dr. Kanaka's revolutionary work at the Madras Institute for Neurology gave their claim to fame [4]; she was the first neurosurgeon in South Asia to introduce these techniques as early as the 1970s.

Social work and other career highlights

In addition to performing thousands of procedures as a physician, Dr. Kanaka wanted to prioritize access to care for lower-income patients in India. During the later stages of her career, Dr. Kanaka set up a cerebral palsy clinic, in partnership with Madras Medical College, to treat patients with cerebral palsy using stereotactic procedures [6]. When delivering care, she made sure to use medical devices that were sourced locally in India and would be a part of a cost-effective system, ensuring that all patients could have the best care even without the highest-quality tools. In honor of her parents, Dr. Kanaka also created the Sri Santhanakrishna Padmavathy Health Care and Research Foundation. The foundation would help provide free healthcare in countries where over 70% of the population was living in poverty [4,6]. Dr. Kanaka loved giving back to the community and has a record in the Limca Book for having donated her blood 139 times to those in need. She helped create deep brain stimulation kits alongside the Indian Biomedical Engineers. Dr. Kanaka was recognized for her work through her extensive research and by associations around the world. In 1996, Dr. Kanaka was the Honorary President of the Asian Women's Neurosurgical Association where she was deemed the first female neurosurgeon of Asia [6].

## Conclusions

Dr. Thanjavur Santhanakrishna Kanaka, a trailblazer in Indian healthcare, distinguished herself through her pioneering contributions to medicine. As a staunch advocate for gender equity in medical education and training, she championed opportunities for women in the field. Her early adoption and advancement of modern surgical techniques in India set her apart from her male contemporaries. Her unwavering dedication to improving patient care and enriching her community in India was matched only by her resilience in overcoming significant obstacles. Her legacy serves as an enduring source of inspiration for generations of physicians, including both male and female neurosurgical trainees. Renowned for her groundbreaking research in stereotactic surgery and electrode implantation, she focused on providing cost-effective care for low-income patients. Outside the operating room, her commitment to service and her efforts to address healthcare disparities in India further highlight her profound impact. Without Dr. Kanaka's remarkable contributions, the field of neurosurgery, and the world at large, would be markedly different.
